# Psychological Stresses in Children Trigger Cytokine- and Kynurenine Metabolite-Mediated Abdominal Pain and Proinflammatory Changes

**DOI:** 10.3389/fimmu.2021.702301

**Published:** 2021-09-01

**Authors:** Kyaimon Myint, Kelly Jacobs, Aye-Mu Myint, Sau Kuen Lam, Yvonne Ai-Lian Lim, Christopher Chiong-Meng Boey, See Ziau Hoe, Gilles J. Guillemin

**Affiliations:** ^1^Department of Physiology, Faculty of Medicine, University of Malaya, Kuala Lumpur, Malaysia; ^2^Neuroinflammation Group, Department of Biomedical Sciences, Faculty of Medicine and Health Sciences, Macquarie University, Sydney, NSW, Australia; ^3^Psychoneuroimmunology Research Group, European Collaborative Project, Munich, Germany; ^4^Department of Pre-Clinical Sciences, Faculty of Medicine and Health Sciences, Universiti Tunku Abdul Rahman, Bandar Sungai Long, Malaysia; ^5^Department of Parasitology, Faculty of Medicine, University of Malaya, Kuala Lumpur, Malaysia; ^6^Department of Paediatrics, Faculty of Medicine, University of Malaya, Kuala Lumpur, Malaysia

**Keywords:** recurrent abdominal pain, stress, neurotrophin, immune mediators, kynurenine pathway

## Abstract

Recurrent abdominal pain (RAP) is a common medically unexplained symptom among children worldwide. However, the biological mechanisms behind the development of functional and behavioral symptoms and changes in blood markers have not been well explored. This study aimed to assess changes in the concentrations of inflammatory markers, including cytokines and tryptophan catabolites, in the serum of children with RAP compared to those with subclinical infections. Children with RAP but without organic diseases were included, and those with asymptomatic intestinal parasitic infections were used as a subclinical infection cohort. Blood samples were collected and used to measure the cytokine profile using Multiplex Immunoassay and tryptophan catabolites using high performance liquid chromatography. Children with RAP showed significantly higher concentrations of serum tumor necrotic factor-α, *p*<0.05, but lower concentrations of IL-10, *p*<0.001, IL-6, *p*<0.001 and brain-derived neurotrophic factors (BDNF) *p*<0.01. In addition, a significant increase in the metabolite of the kynurenine pathway, 3-hydroxyanthranilic acid (3-HAA) *p*<0.01, a significant decrease in the concentrations of anthranilic acid (AA) *p*<0.001, together with an increased ratio of serum 3-HAA to AA (3-HAA/AA) *p*<0.001, was found in this cohort. These findings indicate the significant activation of the immune system and presence of inflammation in children with RAP than those with subclinical parasitic infections. Moreover, children with RAP tested with the *Strengths and Difficulties Questionnaire* (SDQ*)*, displayed high psychological problems though these SDQ scores were not statistically associated with measured cytokines and kynurenine metabolites. We however could hypothesize that the pro-inflammatory state together with concomitant low concentrations of BDNF in those children with RAP could play a role in psychological stress and experiencing medically unexplained symptoms.

## Introduction

In paediatric primary care, children display symptoms that commonly include headache, fatigue and abdominal pain, but only about 10% of these symptoms have an identifiable infectious or metabolic aetiology (1). The term “medically unexplained symptoms (MUS)” is routinely used in paediatric literature to describe those cases (2). MUS or functional symptoms are defined as “somatic symptoms” where no clear infectious or metabolic cause can be identified after comprehensive medical assessment (3, 4). MUS are broad and most of the time, transient and self-limiting.

However, in most MUS cases, the underlying pathophysiology of abdominal pain cannot be identified and is found to be linked with a psychogenic origin. The term “recurrent abdominal pain (RAP)” was used by Apley to refer to the presence of 3 or more discrete episodes of pain over a period of at least 3 months, interfering with normal daily activity (5). RAP prevalence varies, ranging from 10.8% in British school children (5), 10-20% in school-going Singaporean children (1, 6) to 26.9% in Australian students (7). In Malaysia, the overall prevalence of RAP among school children aged from 11 to 16 years was 10.2% (8).

The pathophysiology of RAP and MUS in children is still not well understood. Evidence has shown that these symptoms can be triggered by recent stressful life events (9) and emotional stress (5). It is well established that chronic stress has a significant impact on the hypothalamo-pituitary adrenal (HPA) axis (10) and also on the immune system through inducing the secretion of pro-inflammatory mediators (11, 12). The evidence of stress- induced inflammation is well documented in young adults. Growing up in a socially tough environment (13), childhood abuse (14) and maltreatment (15), and also acute stressful life events (16) are all associated with an increased production of pro-inflammatory cytokines. A concomitant decreased sensitivity of immune cell to anti-inflammatory mediators has also been reported (17). Imbalance between the concentrations of pro-inflammatory- and anti-inflammatory cytokines is associated with the development of psychological illnesses (18–20). Moreover, neurotrophin, brain-derived neurotrophic factor (BDNF), appears to bridge the environmental challenges with enduring change in neuronal function through HPA axis and immune modulation, hence, failure of neuronal adaptive capability may also implicate in development of psychopathological and neurodegenerative diseases (21, 22). When combined, it is likely that a stress-induced immune activation could be associated with the development of symptoms in children with RAP. However, to our knowledge, there is no published literature that has looked at neurotrophin and cytokine responses in children with RAP.

**Figure 1:**
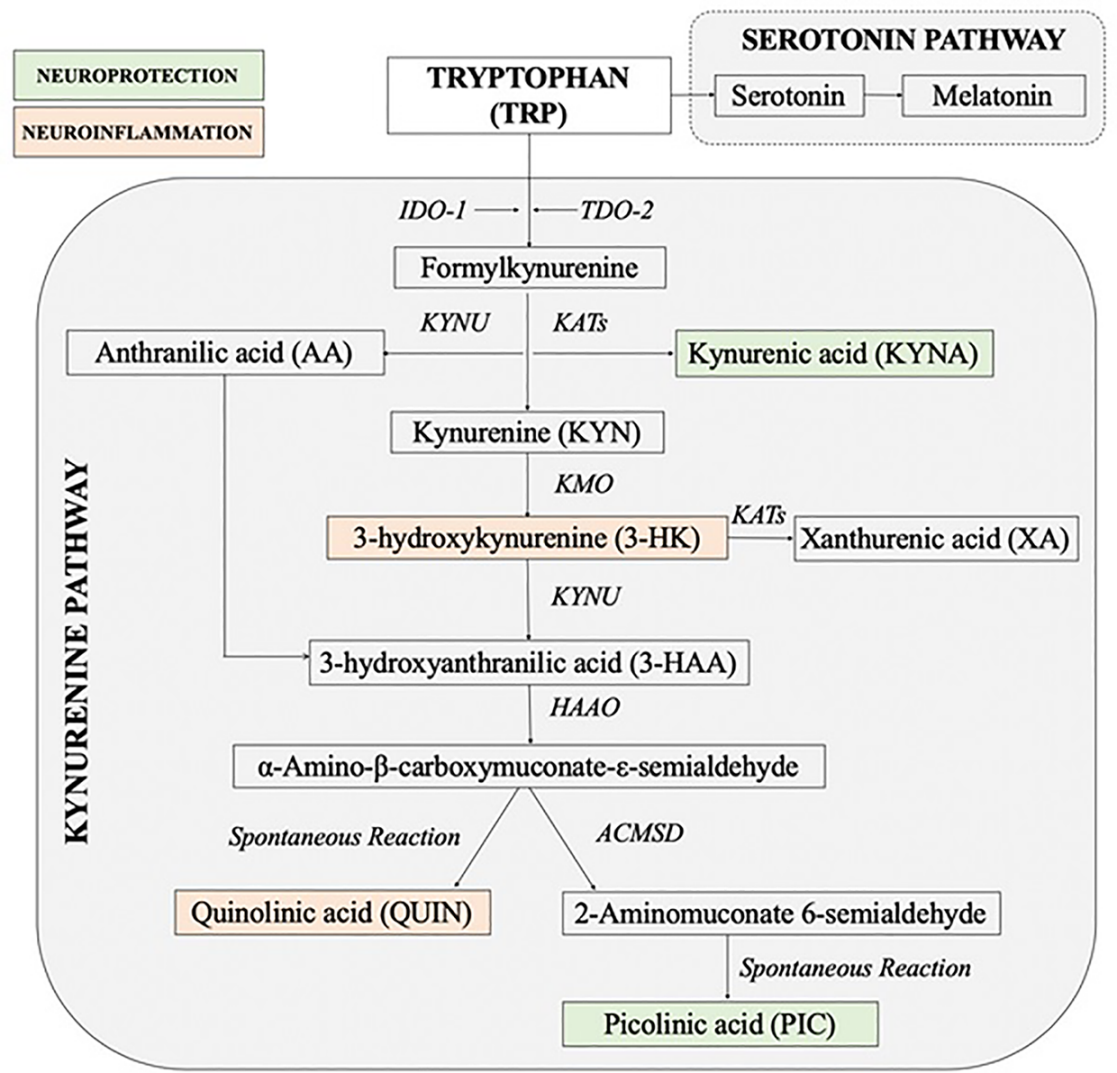
Simplified diagram of the kynurenine pathway [Adapted and modified from (26)]. 3-HAAO, 3-hydroxyanthranilic acid dioxygenase; ACMSD, aminocarboxymuconate semialdehyde decarboxylase; IDO, indoleamine 2, 3-dioxygenase; KATs, kynurenine aminotransferases; KMO, kynurenine 3-monooxygenase; KYNU, kynureninase; TDO, tryptophan 2, 3-dioxygenase.

The kynurenine pathway (KP) of the tryptophan (TRP) metabolism is considered to be a key cross-talk between immune and neuroendocrine systems (23–25). Stress through the increase in cortisol concentrations has a significant impact on the immune system imbalance diverting TRP metabolism away from serotonin and melatonin production towards production of the KP neuroactive metabolites (24, 26–31). The KP can be initiated by activation of indoleamine 2, 3-dioxygenase (IDO-1) in most organs and brain cells including astrocytes, microglia, microvascular endothelial cells and infiltrating macrophages (32) or tryptophan 2, 3-dioxygenase (TDO-2) in liver, kidney and brain (33). While TDO-2 is activated by cortisol, IDO-1 is induced by pro-inflammatory mediators especially interferon-γ (IFN-γ) (31, 34). The key branching point in the KP is the formation of the first stable intermediate kynurenine (KYN) (**Figure 1**). In the context of neuroinflammation, KYN can be converted in 3 ways: 1) by the kynurenine aminotransferases (KATs) into neuroprotective compound kynurenic acid (KYNA) (35, 36) or 2) to neurotoxic metabolites free-radical generator 3-hydroxykynurenine (3-HK), or 3) by kynureninase (KYNU) into anthranilic acid (AA) which can convert to the pro-oxidant and immunomodulatory metabolites 3-hydroxyanthranilic acid (3-HAA). 3-HAA is then metabolized to the excitotoxin quinolinic acid (QUIN) (37). Thus, the involvement of KP in pathophysiology of MUS is highly possible.

The other problem associated with abdominal discomfort in paediatric primary care is intestinal helminth parasitic infection. A large proportion of children infected by parasites are relatively asymptomatic and they belong to the category of subclinical infections. A recent study showed that gastrointestinal nematodes, particularly infections with *Ascaris*, hookworm, and *Trichuris* species can interfere with immune regulation (38). A further study demonstrated that another type of parasitic infection with *Toxoplasma gondii* in animal model could alter the behavior of rodents and induce tryptophan degradation through the KP (39). Toxoplasmosis is associated with the incidence of specific neuropsychiatric conditions in humans (40). Understanding the biological mechanisms behind the complex interactions between parasites and the host immunoregulatory networks to maintain an asymptomatic status during enteric infections are of great interest to researchers and health professionals.

As mentioned above, the dysregulation of the KP through immune-neuroendocrine interactions is implicated in the development of psychological symptoms and neurodegenerative disorders. However, changes in the level of expression of this pathway during chronic stress and subclinical infections have remained unknown. Thus, the main aim of this study is to assess the possible changes in serum levels of neurotrophin, cytokines and KP metabolites in the children with RAP and those with parasitic infections.

## Materials and Methods

### Study Setting and Design

Ethical approval was obtained from the Ethics Committee of the University Malaya Medical Centre (UMMC) Malaysia (MEC Ref. No. 1017.24) prior to the commencement of the study.

### Recruitment of Children With RAP

The children in this study have been attending the outpatient paediatrics clinics at the University of Malaya Medical Centre and University of Malaya Specialist Centre. Complying symptoms of abdominal pain and discomfort were closely monitored in male and female children between 7 to 12 years of age. The children met the RAP criteria set up by Apley (5), (i.e. presence of at least 3 discrete episodes of pain over a period of at least 3 months, interfering with normal daily activity). Patients with no abnormal findings on physical examination, routine blood, urine, and stool tests, abdominal ultrasonographic examination and/or no organic diseases as screened by pediatricians have been considered as children with RAP and included in the study. Strict exclusion criteria were applied for those with abnormal laboratory results, abnormal endoscopy results and any findings suggesting an organic disease. After obtaining written consent from the parent, 10 ml of blood (in a serum-separating tube) was collected from each child between 1630-1830 hr. Blood samples were then centrifuged for 20 min at 2000 rpm at 4°C. Serum was collected, aliquoted and stored at -80°C for quantification of BDNF, cytokines and tryptophan metabolites.

After confirming the absence of organic disease, the mental health status of this cohort of children was further assessed. Children were also requested to complete a validated 25-point Strength and Difficulty Questionnaire (SDQ) to assess their emotional and behavioral problems. This questionnaire is designed to assess the 5 subscales of psychological attributes: emotional symptoms, conduct problems, hyperactivity/inattention, peer relationship problems and prosocial behavior (41, 42). For the younger children, aged 4-10, a Parent Report Measures for Children and Adolescents SDQ (P) 04-10 (English/Malay Language) was used and parents were asked to complete the questionnaires (43). The older children, aged 11 years and above, were asked to complete the Self-Report Measures for Children and Adolescents SDQ (S) 11-17 (English/Indonesia Language) (44).

### Recruitment of Children With Subclinical Infection

Children with asymptomatic parasitic infections were considered as “apparently healthy children with subclinical infections”. As intestinal parasitic infections are highly prevalent among children in the peninsular Malaysian Orang Asli population (45–47), we used samples collected from this community as a part of a cross-sectional study. The original study aimed to determine the prevalence of intestinal helminth infections. The demographic data was obtained and fecal and serum samples collected.

The fecal samples were screened for the presence of ova, cysts, oocysts and parasites. Samples of children 7 to 12 years old that were microscopically positive for either one or more of *Trichuris*, *Ascaris*, hookworm, *Entamoeba, Giardia* and *Hymenolepis nana* infection, and also revealed no history of abdominal pain on screening by questionnaire, were used in this study. Their serum samples were tested for BDNF, cytokines and KP metabolites. Any positive samples from children with a history of gastrointestinal symptoms (abdominal discomfort, abdominal pain, diarrhea, etc.) were excluded from the study.

### Determination of Serum Samples

The concentrations of cytokines, interleukin (IL)-6, IL-10 and tumor necrosis factor-alpha (TNF-α) were quantified using ProcartaPlex multiplex immunoassay (Affymetrix, eBioscience) at the i-DNA laboratory in Kuala Lumpur, Malaysia. The assay was performed according to the manufacturer’s guide, using recommended buffers, diluents and substrates. The endogenous concentration of each individual cytokine analyte was determined based on the respective sample absorbance using a four-parameter logistics curve. The intra-assay coefficient of variation (CV) (calculated as the mean of the CVs for each sample’s duplicate measurements) ranged from 8.36 to 11.42% for all cytokines with limit of detection (LOD) (pg/ml) of 0.34 for BDNF, 0.14 for IL-10, 1.64 for IL-6 and 0.24 for TNF-α.

Serum TRP, KYN, AA, 3-HK and 3-HAA levels were analyzed using ultra high performance liquid chromatography (UHPLC), at the Faculty of Medicine and Health Sciences, Macquarie University, Australia. Concurrent quantification of TRP, KYN and its metabolites were performed based on the method previously described by Guillemin et al. (48) with slight modification (49). An Agilent 1290 infinity UHPLC system, coupled with variable thermostatted volume auto-sampler, the diode-array detector with optofluidic wave guides, fluorescence detector and an Agilent ZORBAX Eclipse Plus C18 reverse-phase column, was used to perform the analytical liquid chromatography. A 0.1 M sodium acetate buffer (sodium acetate 8.2 g in 1 L of ultrapure MilliQ water) with adjusted pH 4.60 (by addition with hydrochloric acid) was used for mobile phase. The concentrations of KYN and 3-HK were quantified spectrophotometrically, at UV absorbance 365 nm with retention time 3.1 and 1.2 minutes, respectively. The TRP, 3-HAA and AA were measured fluorimetrically at specific excitation/emission (**λ**ex/**λ**em) wavelength and retention time (RT); TRP (Ex280 nm/Em438 nm, RT 7.4 min), 3-HAA (Ex320 nm/Em438 nm, RT 3.3 min) and AA (Ex320 nm/Em438 nm, RT 9.8 min). The intra-assay CVs were within the acceptable range of 4-8% for all metabolites. Each measurement was performed in duplicate using deproteinized serum samples. Ten percent trichloroacetic acid (2 g of trichloroacetic acid in 20 mL of ultrapure water) was used to remove the proteins in the samples by precipitation. The targeted different amino acid standards were prepared from respective highest purity (≥98.0%) stock standards (Sigma–Aldrich, Germany). The LOD/limit of quantification (LOQ) were TRP (0.07 uM/0.22 uM), KYN (0.13 uM/0.39 uM), 3-HK (17.79 nM/53.91 nM), 3-HAA (7.06 nM/21.41 nM) and AA (2.48 nM/7.5 nM).

### Statistical Analysis

The SDQ total scores and each subscale score were analyzed according to method used by Goodman (43). In all five subscales of SDQ; emotional problems; conduct disorders; hyperactivity disorders; peer problems and pro-social behaviors; mental health status was predicted as being “probable” (high risk), “possible” (borderline) or “unlikely” (normal).

For the serum parameters, the results were screened for the normality of data by the Shapiro–Wilk test. For normally distributed data, unpaired *t* test with Welch’s correction was used to compare the differences between two independent groups and the results are expressed as mean ± SEM. The Mann-Whitney U test (Wilcoxon two-sample test) was used for those data that were not normally distributed and results are expressed as median and interquartile range (IQR). Correlation between the parameters was analyzed using the Spearman correlation matrix. A *p*-value < 0.05 was considered significant. All statistical analyses were performed using a GraphPad Prism 9.

## Results

### Analysis of SDQ Scores for Children With RAP

Risk of mental health problems as screened by SDQ total scores and each subscale scores are presented in **Table 1**. Twenty and 40% of children are at high and possible risk of mental health problems, respectively.

**Table 1:** Risk of mental health problems as screened by SDQ total scores and each subscale scores in children with RAP.

	% Children with RAP (n=10)		
	Risk of mental health problems		
	Unlikely	Possible	High risk
Total difficulties scale	40	40	20
Emotional symptoms subscale	80	10	10
Conduct problems subscale	60	10	30
Hyperactivity subscale	50	40	10
Peer problems subscale	20	40	40
Prosocial behaviour subscale	90	0	10

### Cytokine Profiles in Children With RAP and Children With Subclinical Parasitic Infections

The concentrations of BDNF and cytokines in the serum samples from the 2 cohorts of children are shown in **Tables 2a–b**. The BDNF concentrations were significantly lower (*p*<0.01) in children with RAP compared to children with subclinical parasitic infections. Higher concentrations of pro-inflammatory cytokines, TNF-α (*p*<0.05) and lower concentrations of anti-inflammatory cytokines, IL-10 (*p*<0.001) were also observed in children with RAP. Meanwhile, the concentrations of IL-6 (*p*<0.001) were higher in children with parasitic infections.

**Table 2:** The BDNF, cytokines and kynurenine profiles in children with RAP and children with subclinical parasitic infections.

	Children with RAP; n= 10	Children with Parasitic Infections (PI); n=17	Ratio RAP/PI	*p* value
	Mean [ ± SEM]^#^/ Median (IQR)^ⱡ^	Mean [ ± SEM] ^#^/ Median (IQR)^ⱡ^		
**a) Concentrations of Neurotrophin**				
BDNF (pg/ml)	540.9 [ ± 127.7]	1252.0 [ ± 87.93]	↓ 2.3	<0.01**
**b) Concentrations of Cytokines**				
IL-10 (pg/ml)	0.37 (0.93-0.08)	2.9 (4.4-1.47)	↓ 7.8	<0.001***
IL-6 (pg/ml)	1.24 (1.24-1.24)	3.91 (9.97-1.63)	↓ 3.2	<0.001***
TNF-α (pg/ml)	0.69 (3.11-0.38)	0.23 (0.95-0.22)	↑ 3	<0.05*
**c) Concentrations of TRP and KP metabolites**				
TRP (µM)	28.46 [ ± 2.67]	25.34 [ ± 1.57]	↑ 1.1	0.2586
KYN (µM)	1.08 [ ± 0.12]	1.19 [ ± 0.09]	↓ 1.1	0.4216
3-HK (nM)	70.77 [ ± 8.13]	58.82 [ ± 3.76]	↑ 1.2	0.1521
3-HAA (nM)	14.47 (15.06-8.18)	3.19 (5.22-2.36)	↑ 4.5	<0.01**
AA (nM)	22.0 (29.76-17.79)	270.0 (330.5-206.2)	↓ 12.3	<0.001***
KYN : TRP	36.39 (41.7-31.31)	47.98 (59.8-41.03)	↓ 1.3	0.05*
3-HAA : AA	0.47 [ ± 0.09]	0.01 [ ± 0.00]	↑ 47	<0.001***

↑Increased; ↓Decreased changes to children with RAP as compared to those with parasitic infections.

^#^Mean [± SEM] for normally distributed data; ^ⱡ^Median (IQR) for not normally distributed data. *p < 0.05; **p < 0.01 and ***p < 0.001.

### KP Profiles in Children With RAP and Children With Subclinical Parasitic Infections

The results of the serum concentrations of the KP metabolites for two groups of children are shown in **Table 2c**. The concentrations of TRP and 3-HK increased in children with RAP but did not significantly change between the groups. However, in children with RAP, we found significantly high concentrations of 3-HAA (*p*<0.01), high 3-HAA : AA ratio (*p*<0.001) and a low AA concentrations (*p*<0.001) and low KYN : TRP ratio (*p*<0.05).

### Correlation Analysis

Correlation analysis showed no significant associations between the concentrations BDNF, cytokines: IL-10, IL-6, TNF-α and KP metabolites in both cohorts of children. In addition, there was no association between SDQ scores and tested biochemical parameters in children with RAP.

## Discussion

The medically unexplained or functional somatic symptoms constitute a major clinical problem in paediatric primary care (3, 4), however, the mechanisms behind the occurrence of these symptoms have not yet been addressed. Thus, this study is aimed to access the changes in serum levels of neurotrophins, cytokines and KP metabolites in the children with RAP and those with parasitic infections. As evidenced by previous reports (9, 13, 14, 16), psychological stress appeared to be a potential candidate accounting for occurrence of unexplained or functional somatic symptoms, thus the cohort of children with RAP were assessed by SDQ to assess their emotional and behavioral status. As compared to normative SDQ data on total difficulty scores (43), 60% of children with RAP were found to be at risk of getting mental health problems. SDQ-symptoms scores were also interpreted and “caseness” from symptoms scores were further defined according to normative data provided in the manual (43). Thirty-and 40% of children were at high risk of conduct and peer problems respectively while 10% of children showed abnormal emotional, hyperreactivity and prosocial behavior problems. As SDQ is used clinically and appears the best-suited test to identify the psychological problems in both children and adolescents (41, 42), we confirmed that children with RAP strongly imply the presence of severe psychological problems.

These borderline and abnormal total difficulties as well as symptoms scores are likely to be associated with significantly lower concentrations of BDNF in children with RAP. The BDNF is an essential mediator of neuronal plasticity and is expressed throughout the brain, with higher abundance in areas controlling cognition, mood and emotion such as the hippocampus and cerebral cortex (50). Although the peripheral sources of BDNF remain unclear, this neurotrophin is found in large amounts in platelets (51). This finding on BDNF is in accordance with previously published studies that showed stress was associated with down regulation of BDNF production (52, 53) and high activity of glucocorticoids receptors (54). Patients with depressive disorders have significantly lower concentrations of BDNF in their blood (22, 55) and interestingly, after antidepressant treatments, BDNF concentrations were back to physiological concentrations (52, 55). We hypothesize that the low concentrations of BDNF in the children with RAP could trigger brain biochemical dysfunction and ultimately, lead to the development of psychopathological symptoms.

The results of cytokine profiles showed interesting findings. The concentrations of TNF-α, proinflammatory cytokines, are expected to be increased in infection; however, its concentrations were significantly increased in children with RAP than that with parasitic infection. This may be explained by repeated and chronic psychological stress-induced pro-inflammatory cytokines production (11, 15, 56, 57). The inflammatory markers, IL-1β and TNF-α are the most consistently reported cytokines responsible for psychological stress response regardless of stressors and species (11, 58). The lower concentrations of TNF-α in this study cohort of children with parasitic infections may be due to their significantly higher concentrations of IL-10. IL-10, a cytokine with broad immunoregulatory function, is known to inhibit the production of iNOS, IFN-γ, IL-12, TNF-α production and suppresses parasite killing in a variety of protozoan and helminth infections (59, 60). In addition, these cohort of children also showed increased concentrations of a pleiotropic cytokine, IL-6. This may be due to its immunosuppressive effect as well as regenerative effect as IL-6-dependent mucosal protection was demonstrated in enteric bacterial pathogen (61). Together, the impact of IL-10 and IL-6 modulated immunosuppression is found to be beneficial and could contribute the apparently healthy status in them (60).

No significant changes were observed in TRP and KYN concentrations between the 2 groups. However, lower (downstream) KP metabolites showed significant changes. We found higher concentrations of 3-HK (1.2 folds - not statistically significant), and significantly elevated 3-HAA concentrations (4.5 folds) in RAP children. In contrast, the concentrations of AA were significantly decreased (12.3 folds) in RAP children. This can be explained by a higher activity of the KMO enzyme directing KYN catabolism towards the production of 3-HK instead of AA. 3-HK is then catabolized by KYNU to produce 3-HAA. This shows that the KP is moving towards its inflammatory/neurotoxic branch which is highlighted by the high increase in the 3-HAA/AA ration (47 folds).

3-HAA is a free-radical generator, producing hydrogen peroxide and superoxide promoting oxidative protein damage (62, 63) and inducing apoptosis (64). 3-HAA can also have excitotoxic effects (65). More recently, its immune modulatory functions have been demonstrated (66). 3-HAA is also the main precursor for biosynthesis of the excitotoxin QUIN (67). It is likely that, in children with RAP, the KP is shifted to the neurotoxic branch leading to increased QUIN production. Existing literature has documented the involvement of 3-HAA in the initiation, development and amplification of neurodegenerative processes (27, 68–70). Unfortunately, the concentrations of PIC and QUIN (67, 71) have not been measured and represent the limitations of this study. These parameters could have tightened the association with the KP activation and psychopathological symptoms (72). As we previously published, QUIN has been identified as a key player in depression (73) and suicide (72).

In addition, the ratio of serum 3-HAA to AA (3-HAA/AA) significantly increased in children with RAP. This indicates the lack of a neuroprotective response, likely due to the lack of KYNA, since a decreased 3-HAA/AA ratio is found in many neuroinflammatory conditions and associated with a loss of anti-inflammatory response (74). It is well documented that the activation of KP is controlled by inflammatory mediators especially cytokines. The initial step in the KP, conversion of TRP to KYN, is regulated by enzyme IDO-1, which is induced by pro-inflammatory cytokines such as IFN-γ, IL-1β and also IL-6 (23, 75, 76). This is not surprising as they are not the cytokines known to be involved in KP activation except for IL-6. However, the interesting results were the significant higher concentrations of pro-inflammatory TNF-α- and lower concentrations of anti-inflammatory IL-10- cytokine in the RAP children without organic disease compared to children with parasitic infections. It should be emphasized that stress can trigger immune activation, and, even more importantly, contribute to the induction of pro-inflammatory cytokine, TNF-α. However, an increased kynurenine/tryptophan (KYN/TRP) ratio in children with parasitic infection may indicate the possibility of other cytokine induced IDO-1 activation, especially IFN-γ, which has not been quantified in this study. The fact that there are no changes in TRP, KYN, a decreased KYN/TRP ratio, high concentrations of 3-HAA and low concentrations of AA clearly implies that the enzyme kynurenine monooxygenase (KMO) is strongly activated and could represent a relevant therapeutic target for children with RAP.

There are some limitations in the present study. In this study, we did not find any correlation between the concentrations of the inflammatory cytokines and KP metabolites. We neither found statistical association between SDQ scores and biochemical data. This indicates the needs of further study with larger sample size to find out the associations between SDQ data and inflammatory markers and KP metabolites. As the study population is small, the results obtained cannot be extrapolated to the general population. In addition, it would be of interest in the future to determine the KP profile in normal healthy children although it is hard to get the samples due to ethical concerns. Furthermore, as stated above, the quantification of the late KP metabolites of QUIN and PIC, are lacking in this study.

To conclude, this study is the first study to examine the involvement of the KP, inflammatory cytokines and BDNF in children with RAP. Our results strongly suggest that the decrease in BDNF concentrations and concomitantly, the production of the neuroactive KP metabolites, 3-HAA, and, to a lesser extent, 3-HK, may lead to the alteration of physiological processes and possibly explain the emergence of psychological symptoms in these children. A future longitudinal study with a larger cohort would be critical to validate our pilot study to better understand the complex interplay between chronic stress, cytokine networks, and KP dynamics in psychological processes in RAP children. The wider significance of this study lies in the fact that it provides evidence to shed light on the possible mechanisms for mind-body interactions that are increasingly observed and recognized in clinical practice.

## Data Availability Statement

The raw data supporting the conclusions of this article will be made available by the authors upon request.

## Ethics Statement

The studies involving human participants were reviewed and approved by University Malaya Medical Centre (UMMC) Malaysia (MEC Ref. No. 1017.24). Written informed consent to participate in this study was provided by the participants’ legal guardian/next of kin.

## Author Contributions

AM, SH, SL, and KM conceptualized the study. YL and CB contributed in sample collection. GG designed the experiments. KM and KJ performed the assays and analysis. KM wrote the manuscript. SH, YL, CB, SL, and GG edited the manuscript. All authors contributed to the article and approved the submitted version.

## Funding

The work of this study is fully funded by University of Malaya Research Grant, UMRG 489/12 HTM. GG is supported by the National Health and Medical Research Council (NHMRC), the Australian Research Council (ARC) and Macquarie University.

## Conflict of Interest

The authors declare that the research was conducted in the absence of any commercial or financial relationships that could be construed as a potential conflict of interest.

## Publisher’s Note

All claims expressed in this article are solely those of the authors and do not necessarily represent those of their affiliated organizations, or those of the publisher, the editors and the reviewers. Any product that may be evaluated in this article, or claim that may be made by its manufacturer, is not guaranteed or endorsed by the publisher.
